# "Pseudosarcoma" in a pregnant woman

**DOI:** 10.1186/1477-7819-5-7

**Published:** 2007-01-18

**Authors:** Amarjit Anand, Eva Maria Tsapakis, Ali A Narvani, Ali Alhakim, Steve R Cannon, Eleftherios Tsiridis

**Affiliations:** 1 Royal Free and University College London Hospitals, Royal National Orthopaedic Hospital, Bone Tumour Unit, Brockley Hill, Stanmore, Middlesex, HA7 4L, UK

## Abstract

**Background:**

Intravascular fasciitis (IVF) is a rare benign condition characterised by reactive myofibroblastic proliferation arising from the superficial or deep fascia and involving arteries and/or veins. It is a distinct variant of the more common condition of nodular fasciitis, which possesses similar clinical and histological features to IVF, but lacks vascular invasion. A thorough review of the literature revealed 26 reported cases of IVF.

**Case presentation:**

We report a case of IVF in a 16-week pregnant lady affecting the hypothenar eminence of the hand associated with the ulnar artery.

**Conclusion:**

The characteristic involvement of muscular arteries and veins by reactive myofibroblastic proliferation in IVF suggests a malignant component and often leads to an inappropriate diagnosis for this benign condition. We propose that hormone-related changes associated with pregnancy may play an important role in the aetiopathogenesis of this myofibroblastic lesion.

## Background

Intravascular fasciitis (IVF) is a term originally described by Patchefsky and Enzinger to describe this distinctive variant of nodular fasciitis [1]. It is a benign, reactive myofibroblastic proliferative lesion that arises from the superficial or deep fascia and involves predominately small to medium-size arteries and/or veins. It presents as a well-defined nodule in the subcutis or muscle. The involvement of muscular vessels can lead to an erroneous assumption of malignant vascular invasion. It is considered to be very rare and since its first description in 1981, only a few isolated cases have been documented in the literature [2-8].

## Case Presentation

In August 2003, a 20-year-old 16-week pregnant mother of one, presented to the orthopaedic outpatient clinic with a 2-month history of a slowly growing painless mass located in the right hypothenar eminence. She was right-hand dominant and a housewife. There was no history of trauma, insect bites or drainage from the area. There was no significant past medical or surgical history. She was not on any prescribed medication. There was no family history of tumours. She denied smoking, alcohol and drug abuse. On examination, a non-tender, firm to palpation, well-demarcated and tethered to the subcutaneous tissues mass, surrounded by mild erythema, and measuring approximately 2.5 cm × 3.0 cm was revealed, in the absence of palpable lymph nodes at the ipsilateral elbow or axilla. There were no other abnormal signs.

**Figure 1:**
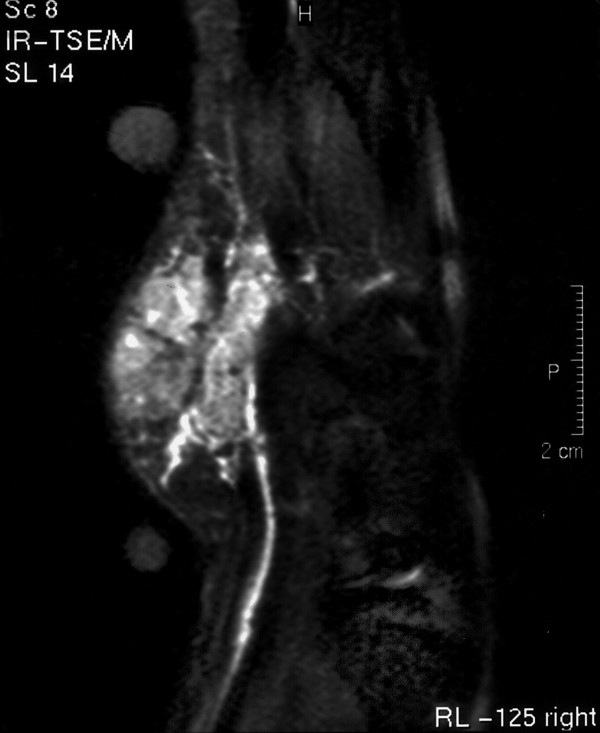
MRI of the right hand showing a pseudosarcomatous mass encircling the ulnar artery.

Haematological and biochemical investigations were within the normal range. A plain X-ray of the right hand and forearm was unremarkable. Magnetic resonance imaging showed the mass to have infiltrated all skin layers and to have encircled the ulnar artery without involvement of the underlying bones (Figure 1). The absence of a signal void from within the mass excluded a diagnosis of haemangioma. Ultrasound-guided needle biopsy showed lesional areas consistent with collagenous fibrous tissue of varying cellularity, with little evidence of mitotic activity. Immunohistochemistry showed focal positivity for smooth muscle actin (SMA), suggesting myofibroblastic differentiation. Histological appearances were in keeping with fibromatosis.

**Figure 2:**
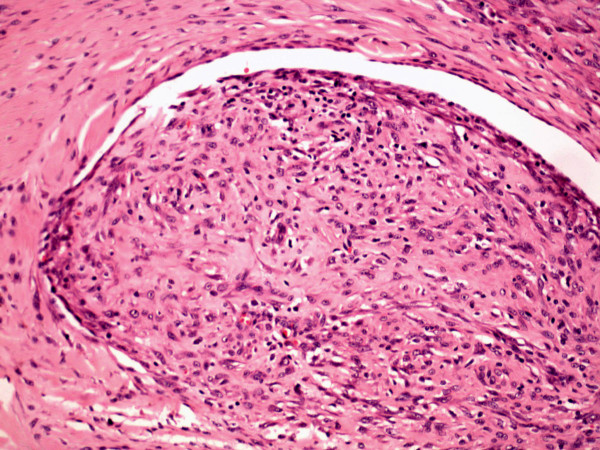
Section demonstrating an intravascular proliferation of uniform spindle cells. (H&E ×10).

Three weeks after initial presentation, the lesion was excised preserving the ulnar nerve and artery. Histology showed a multi-nodular, well-circumscribed uniform spindle cell lesion with varying cellularity and fibrosis. In one section, central cyst formation was observed. Occasional mitoses of normal morphology were seen. Necrosis was not a feature. In several areas, the lesion appeared to be intravascular, the vessel was lined by an apparent endothelium (Figure 2). Scattered osteoclast-like multi-nucleate giant cells were also observed. Immunohistochemistry replicated diffuse positivity for SMA, but not for desmin, CD34 or S100 protein. Appearances were consistent with a myofibroblastic proliferation, confirming a diagnosis of intravascular fasciitis (IVF).

The patient had an uneventful recovery and required no further therapy. She continues to be evaluated for local recurrence every 6 months, with no sign of recurrence at the 2-year follow-up.

## Discussion

IVF was first described as a subset of cases closely resembling the clinical and histological picture of nodular fasciitis (NF) [1]. Recognition of IVF as a variant of NF rather than as a vascular neoplasm, or as a low-grade sarcoma, which demonstrates local infiltration of normal tissue and intravascular involvement, is necessary to avoid over-diagnosis and over-treatment of this benign myofibroblastic lesion. IVF originates from fibroblasts associated with arterial or venous walls, and is thought to represent reactive overgrowth of normal cells without malignant transformation. Histologically, IVF is characterised by fibroblasts arranged in fascicles and bundles, with oval, pale-stained nuclei and occasional prominent nucleoli. Typical mitotic figures may be present. The intracellular matrix is rich in mucopolysaccharides, with extravasated erythrocytes, foamy macrophages, and occasional multinucleated giant cells present [8,9].

Pre-existing trauma, viral infection, and venous thrombosis resulting in myofibroblastic transformation of the vessel wall have all been implicated in the aetiopathogenesis of IVF, but there was no evidence for either of these in our case affecting the hypothenar eminence. Pregnancy was the only significant co-existing medical condition.

We hypothesised that hormone-related changes seen during pregnancy may have contributed to the development of IVF in our patient. Oestrogen in particular, has for long been known to stimulate fibroblast and smooth muscle cell types and has been implicated in fibroproliferative diseases such as carpal tunnel syndrome, Dupuytren's contracture, and dermoid tumours. A weak presence of oestrogen receptors has also previously been demonstrated in nodular fasciitis [10]. Oestrogen-related proliferation of fibroblasts and smooth muscle cells is very rapid in early pregnancy due to higher oestrogen levels and decreases progressively thereafter [11].

## Conclusion

IVF is a benign myofibroblastic proliferative lesion that involves arteries and/or veins, a feature distinguishing it from other pseudosarcomatous proliferative lesions. Nevertheless, it may be easily mistaken for a malignant sarcomatous invasion of the vasculature. Based on our case presentation, we propose that pregnancy and oestrogen-related changes may be another risk factor for IVF and play an important role in the aetiopathogenesis by influencing proliferative changes in fascia myofibroblasts, however, more research and further cases need to be studied prior to establishing such hypothesis.

## Competing interests

The author(s) declare that they have no competing interests.

## Authors' contributions

AA^1 ^reviewed the literature and prepared the manuscript. SRC^5 ^provided the case and revised the manuscript. AN^3 ^obtained patient consent and helped in preparation of the manuscript. ET^6 ^and EMT^2 ^conception and identification of case hypothesis, critical revision of the manuscript and coordination of the study. AA^4 ^provided the histology images and histology information. All authors read and approved the final manuscript.
